# Supplementary material for: The interactome of intact mitochondria by cross-linking mass spectrometry provides evidence for coexisting respiratory supercomplexes
Source: Mol Cell Proteomics. 2017 Dec 8;17(2):216–32. doi: 10.1074/mcp.RA117.000470 (PMC5795388; doi:10.1074/mcp.RA117.000470)
Supplement: Supplemental Data [file supp_RA117.000470_133922_0_supp_23978_fzffwf.zip › spectra_annotation/mito_DR_spectra_annotation/181-1-10-1-3-1.pdf]

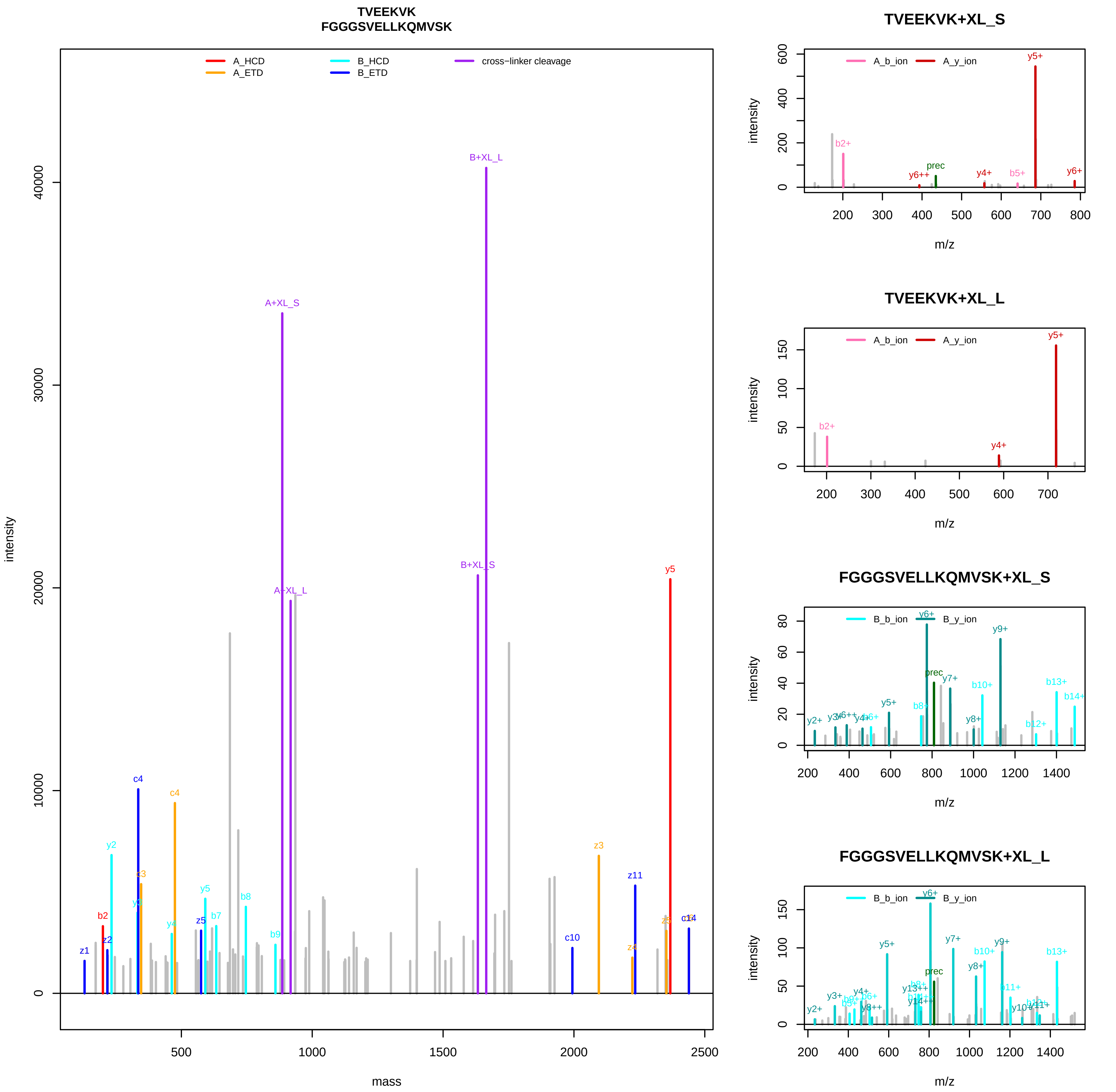
TVEEKVK
FGGGSVELLKQMVSK
A_HCD
A_ETD
B_HCD
B_ETD
cross−linker cleavage
intensity
mass
B+XL_L
A+XL_S
B+XL_S
A+XL_L
y5
c4
c4
z3
z11
y2
c3
b2
z2
z1
y3
y4
z5
y5
b7
b8
b9
c10
z4
c14
TVEEKVK+XL_S
A_b_ion
A_y_ion
y5+
b2+
prec
y6++
y4+
b5+
y6+
m/z
TVEEKVK+XL_L
y4+
FGGGSVELLKQMVSK+XL_S
B_b_ion
B_y_ion
y6+
y9+
y7+
b10+
b13+
b14+
y8+
b12+
b8+
FGGGSVELLKQMVSK+XL_L
y7+
y9+
b11+
